# Cancer-Related Functions and Subcellular Localizations of Septins

**DOI:** 10.3389/fcell.2016.00126

**Published:** 2016-11-08

**Authors:** Christian Poüs, Laurence Klipfel, Anita Baillet

**Affiliations:** ^1^Institut National de la Santé et de la Recherche Médicale, UMR-S 1193, Université Paris-Sud, Université Paris-SaclayChâtenay-Malabry, France; ^2^Laboratoire de Biochimie-Hormonologie, Hôpital Antoine Béclère, AP-HPClamart, France; ^3^Département de Génétique, Institut de la Vision, Université Pierre et Marie Curie Paris 06, Sorbonne Universités, Institut National de la Santé et de la Recherche Médicale UMR-S 968, Centre National de la Recherche Scientifique UMR 7210Paris, France

**Keywords:** septin, cancer, plasma membrane, actin cytoskeleton, microtubules

## Abstract

Since the initial discovery of septin family GTPases, the understanding of their molecular organization and cellular roles keeps being refined. Septins have been involved in many physiological processes and the misregulation of specific septin gene expression has been implicated in diverse human pathologies, including neurological disorders and cancer. In this minireview, we focus on the importance of the subunit composition and subcellular localization of septins relevant to tumor initiation, progression, and metastasis. We especially underline the importance of septin polymer composition and of their association with the plasma membrane, actin, or microtubules in cell functions involved in cancer and in resistance to cancer therapies. Through their scaffolding role, their function in membrane compartmentalization or through their protective function against protein degradation, septins also emerge as critical organizers of membrane-associated proteins and of signaling pathways implicated in cancer-associated angiogenesis, apoptosis, polarity, migration, proliferation, and in metastasis. Also, the question as to which of the free monomers, hetero-oligomers, or filaments is the functional form of mammalian septins is raised and the control over their spatial and temporal localization is discussed. The increasing amount of crosstalks identified between septins and cellular signaling mediators reinforces the exciting possibility that septins could be new targets in anti-cancer therapies or in therapeutic strategies to limit drug resistance.

## Introduction

In humans, 13 septin proteins have been identified, which can be clustered into four groups according to their gene structure homology (Kinoshita, 2003; Hall et al., 2005). A plethora of isoforms has been described, and for SEPT4, 8 and 9, long and short N-terminal extensions have been identified (see Hall and Russell, 2012). Such diversity allows a large repertoire of septin assemblies, which could explain the multiplicity of septin subcellular localizations and functions. Septins can oligomerize into repeated and ordered hexamers or octamers, which can organize into higher-order structures, including rings or filaments. Ring structures have been well described in the context of mitotic completion or in differentiated cell structures like at the base of primary cilia or in the spermatozoid annulus (for reviews, Caudron and Barral, 2009; Saarikangas and Barral, 2011; Spiliotis and Gladfelter, 2012). Septins are also observed as rods and filaments, which associate with actin, microtubules (MTs) or membranes and are thus considered as a fourth component of the cytoskeleton (Mostowy and Cossart, 2012). Septins function as scaffolds for protein-protein interactions, or as diffusion barriers for protein compartmentalization, not only during cell division, but also in an increasing number of processes in interphase cells (for reviews, Mostowy and Cossart, 2012; Fung et al., 2014; Montagna et al., 2015).

Septins have initially been identified as fusion partners with MLL in leukemia (Osaka et al., 1999; Cerveira et al., 2011). Their gene expression is also deregulated in tumors. *SEPT2, 8, 9*, and *11* genes are consistently up-regulated, while *SEPT4* and *10* are down-regulated in many cancer cells (Liu et al., 2010; Montagna et al., 2015). A colon cancer diagnosis method based on the quantification of circulating methylated *SEPT9* DNA has even been proposed (for review, Song and Li, 2015). Septin isoform expression (mainly focused on SEPT9 isoforms) has also been studied in a broad range of solid tumors (Scott et al., 2006; Connolly et al., 2011, 2014; Shen et al., 2012; Gilad et al., 2015). Here, we focus on the links between the modulation of septin polymer composition, their differential subcellular localization, and the molecular and cellular pathophysiological mechanisms they affect in cancer, both in mitotic and interphase cells.

## Roles of septins in mitosis

By forming highly organized rings at cell division sites, septins have been found to play a crucial role in the spatio-temporal control of yeast budding, and the mechanisms that control septin assembly, remodeling and functions in this context are still thoroughly explored in the yeast model. In mammalian cells, septins have also been identified as one of the contributors of mitosis, and could potentially be implicated in a variety of cancers. Indeed, after Cdk1-mediated phosphorylation, long SEPT9 isoforms become a substrate of the prolyl-isomerase Pin1, and their isomerization is required for cytokinesis completion (Estey et al., 2013). Like other oncogenes and tumor suppressors controlled by Pin1 (for review, Zhou and Lu, 2016), specific SEPT9 isoforms may thus participate in oncogenesis. Also, septins contribute to fulfill proper chromosome congression and correct segregation during the anaphase. In this context, the SEPT2/6/7 complexes seem to be important for the recruitment of the kinesin family protein CENP-E (Spiliotis et al., 2005), which participates in the mitotic checkpoint, and for chromosome movement along MTs during the anaphase. At the onset of telophase, septins concentrate at the central spindle region where they interact with the actomyosin contractile ring via the partner protein anillin (for reviews, Fung et al., 2014; Menon and Gaestel, 2015). Recent data indicate that the anillin-septin ring promotes the intercellular bridge ingression, elongation and narrowing. These steps occur prior to septin and anillin relocalization to the central stem body and to sites of MT constriction. There, the septin ring facilitates the recruitment of Chmp4B, allowing the assembly of the ESCRT III complex, which actually mediates the abcission step (Renshaw et al., 2014). A recent study on the effects of chrysotile fibers (responsible of mesothelioma, lung cancer, and asbestos) points out the role of cytokinesis failure mediated by an overexpression of SEPT2 and by anillin and SEPT9 mislocalizations, in causing aneuploidy, centrosome amplification, and multipolar mitoses (Cortez et al., 2016), which are frequent in cancer cells.

## Localization-dependent roles of septins in interphase cells

**Figure 1:**
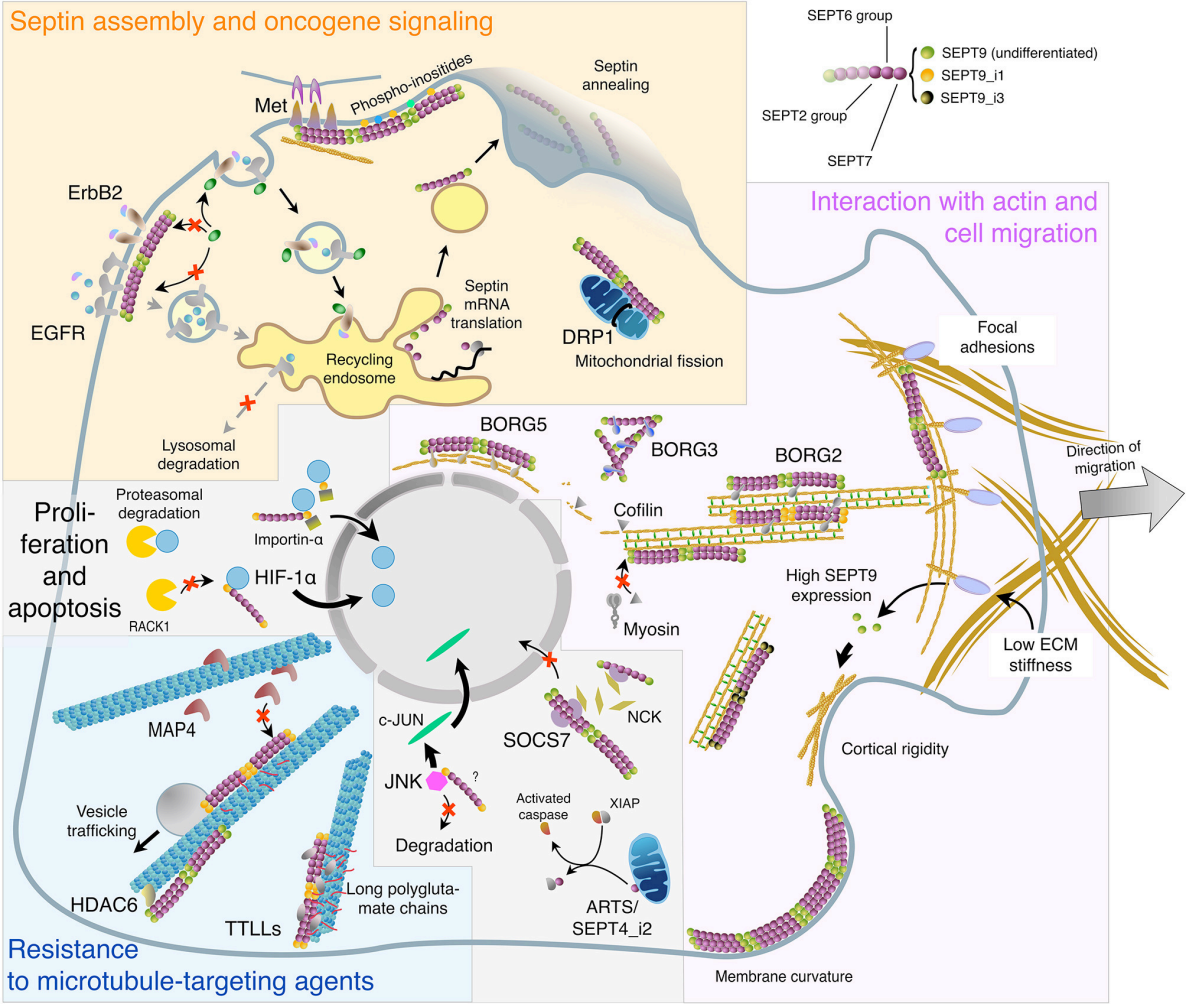
**Subcellular localizations and functions of septins in interphase cells in relation with oligomer composition and binding partners**. This virtual cell summarizes the main topics described in the text regarding: Septin biosynthesis, annealing, and association with the plasma membrane. Role of septin filaments in oncogene receptor signaling and dynamics, and in mitochondria fission (Yellow panel). Septin localization to the actin cytoskeleton: direct binding or involvement of SEPT9 isoforms and BORG proteins. Implication of septin filaments in actin filament bundling and organization, cell migration, cortical rigidity, and membrane curvature sensing (Magenta panel). Septin binding to microtubules and the links with tubulin post-translational modifications and MAPs in vesicular trafficking and in cell resistance to microtubule-targeting agents (Cyan panel). Septin roles in the nuclear import of signaling factors involved in cell proliferation and in apoptosis. It is not known whether SEPT9 interacts alone or as part of septin oligomers with HIF-1α, importin-α and JNK (Light gray panel). The color code of septin monomers is indicated on the top right of the figure.

Out of the cell division context, septin contribution to cancer may also involve interphase cells, in a way that is linked to their subcellular location, as described hereafter and summarized in Figure 1.

### Membrane-associated septins

Septin self-assembly into filaments and higher-order structures occur by diffusion-driven annealing on the plasma membrane (Bridges et al., 2014). In return, large septin filament arrays stably interacting with the plasma membrane may modify cortical morphogenesis by imposing membrane curvature (Tanaka-Takiguchi et al., 2009), and affect the cortical rigidity of migrating cells (Tooley et al., 2009), thus contributing to tumor metastasis.

Besides their role in membrane compartmentalization, septins have been implicated in the misregulation of growth factor receptors involved in cancer progression. They can cluster and stabilize plasma membrane proteins (Caudron and Barral, 2009; Hagiwara et al., 2011) including receptor tyrosine kinases. Indeed, membrane-associated SEPT9 prevents CIN85 binding to the ubiquitin ligase Cbl, resulting in reduced ubiquitin-dependent EGFR degradation (Diesenberg et al., 2015). Also, septins are involved in the abnormal persistence of ErbB2 at the plasma membrane of cancer cells via decreased ubiquitylation and degradation (Marcus et al., 2016). In addition, the surface distribution of the c-Met protooncogene is regulated in opposite ways by SEPT2 and 11, but both participate in its interaction with the ligand and anchorage to the actin cytoskeleton (Mostowy et al., 2011), illustrating the importance of the subunit composition of septin filaments in controlling their biological functions.

Septins may also associate with other membrane-bound organelles. SEPT2 and 7, by interacting with DRP1 would concentrate it at the sites of mitochondrial constriction and facilitate their fission (Pagliuso et al., 2016; Sirianni et al., 2016). Mitochondria-associated septins also comprise the isoform 2 of SEPT4 (SEPT4_i2) also called ARTS, which upon pro-apoptotic stimuli, is released in the cytosol where it binds to the XIAP proteins to release the inhibition of caspases and thus promote apoptosis (Edison et al., 2012). ARTS expression has been shown to drop in acute lymphoid leukemias and in lymphomas, and would be implicated in controlling the number of normal stem cells (García-Fernández et al., 2010). This has led to propose ARTS as a valuable therapeutic target against cancer stem cells (Elhasid and Larisch, 2011). Also, septins are implicated in autophagosome formation upon nutrient deprivation in yeast (Barve et al., 2016). These studies provide new clues to understand the role of septins in cancerogenesis and/or adaptation to tumoral environment.

### Actin-associated septins

In many cell types, septin filaments coalign with actin in subcortical regions or along stress fibers (Kinoshita et al., 1997). Actin loss causes septins to form free cytoplasmic rings (Kinoshita et al., 2002). Conversely, SEPT2 depletion attenuates actin bundling (Kinoshita et al., 2002) and disrupts stress fibers (Schmidt and Nichols, 2004). Knocking down SEPT6 and 7 (Kremer et al., 2007) produce a similar loss of actin bundling along with the disruption of cell polarity. These effects may involve septin binding partners that crosslink septins to actin like BORG2 and BORG5, which are two of the five Cdc42-effector proteins of the BORG family (Liu et al., 2014; Calvo et al., 2015). Stress fiber disruption may also involve septin partners that directly regulate actin like the protein Wdpcp (Cui et al., 2013).

Septins control actin remodeling during cell migration and may thus contribute to metastatic cancer cell dissemination and invasion. Septin filaments bind to and stabilize the transverse arc and radial stress fibers in the lamellipodia of migrating cells (Dolat et al., 2014). Such stabilization could involve SEPT9-mediated prevention of actin depolymerisation by myosin and cofilin (Smith et al., 2015). Septins also contribute to the stabilization of nascent focal adhesions (Dolat et al., 2014), which is necessary for their turnover and thus for effective migration. *Sept9*-knockout mouse embryonic fibroblasts indeed migrate more slowly than wild-type cells (Füchtbauer et al., 2011). Also, SEPT9_i4 is involved in the control of migration directionality in MCF7 breast cancer cells (Chacko et al., 2005). Persistent directional migration is further dependent on SEPT7 and on BORG5, which maintain proper actin filament organization (Liu et al., 2014).

Cell migration and invasion requires epithelial-mesenchymal transition (EMT), which involves the formation of cell protrusions and changes in the way cells interact with the extracellular matrix (ECM). Depletion of SEPT9 in various metastatic cancer cells allows the reversion of EMT and reduces cell spreading, migration and invasion (Shankar et al., 2010). SEPT1 also participates in the spreading of squamous cell carcinoma DJM-1 cells (Mizutani et al., 2013).

Tumor progression also requires neoangiogenesis, which implies the migration of leader cells of the tumor microenvironment. Interestingly, septins are involved in both processes. Indeed, Yeh et al. (2012) proposed that the ECM stiffness controls SEPT9 expression of endothelial cells via integrin signaling, and regulates cell proliferation and peripheral distribution of actin assembly. Regarding tumor-associated fibroblast migration, Calvo et al. (2015) showed that the cohesion between actin fibers and septins is increased due to elevated expression of the crosslinking protein BORG2. This in turn leads to matrix remodeling, favors the activation of highly contractile cancer-associated fibroblasts and promotes cancer cell invasion, angiogenesis, and tumor growth.

### Microtubule-associated septins

In a few cell types, septin filaments co-align with MTs (Surka et al., 2002; Nagata et al., 2003; for review, Silverman-Gavrila and Silverman-Gavrila, 2008). They compete with the MT-stabilizing protein MAP4 to associate with the MT lattice and reduce MT stability (Kremer et al., 2005; Spiliotis et al., 2008). By contrast, septin-decorated MTs also exhibit lower MT dynamics in MDCK cells (Bowen et al., 2011) although septins do not colocalize with stabilized acetylated or detyrosinated MTs (Spiliotis et al., 2008). However, the relationship between septins and MT acetylation is still unclear as, in dendrites, SEPT7 was found to interact with the tubulin deacetylase HDAC6 (Ageta-Ishihara et al., 2013). In addition, septin filaments interact with polyglutamylated MTs and favor vesicle trafficking along these tracks to maintain the polarity of MDCK cells (Spiliotis et al., 2008).

Cancer chemotherapy often makes use of MT-targeting agents (MTA), which do not only act during mitosis but also interfere with MT dynamics during the interphase. MT-associated septins by modulating the MT environment may therefore modulate MT-based activities. Several septins have been proposed to participate in cancer cell resistance to MTA. Low SEPT10 expression level would promote paclitaxel resistance (Xu et al., 2012), while resistance to paclitaxel and to 2-methyl-estradiol involves SEPT9_i1 overexpression in several cancer cell lines (Amir and Mabjeesh, 2007). Also, misregulations of SEPT9_i1 and i4 have been linked to bad prognosis and resistance to MTA in prostate (Gilad et al., 2015) and breast cancers (Chacko et al., 2012). Consistently, paclitaxel-resistant MDA-MB 231 breast cancer cells display increased SEPT2, 8, 9, 11 levels (Froidevaux-Klipfel et al., 2011). In these cells, septins are displaced from actin fibers to MTs, where they restore higher level of MT dynamics by acting as scaffolding proteins to recruit tubulin polyglutamylation enzymes. Septin recruitment to polyglutamylated MTs result in increased binding of MT modulators that play key roles in controlling catastrophe and rescue events (Froidevaux-Klipfel et al., 2015).

Septins not only form a diffusion barrier at the base of the primary cilium (Hu and Nelson, 2011), but also associate with acetylated MTs in the axonema of RPE-1 cells in which the SEPT2/7/9 complex controls ciliary length (Ghossoub et al., 2013). The loss of TTLL3 activity, a polyglycylase required for robust primary cilium formation has been involved in colon cancer development (Rocha et al., 2014). By analogy with the finding that septin filaments recruit tubulin polyglutamylases on MTs (Froidevaux-Klipfel et al., 2015), the axonema-associated septins could perhaps function to scaffold TTLL3 on ciliary MTs, as it belongs to the same family of enzymes.

### Other roles of septins in ill-defined locations

As observed for membrane receptors, septins, and more precisely SEPT9_i1, stabilize other signaling proteins like JNK (Gonzalez et al., 2009) or HIF-1α (Amir et al., 2009) by preventing their degradation. However, these studies used total cell extracts and gave no indication of where these events take place in the cell. In both cases, it is not known whether septins function as filaments. By stabilizing JNK, SEPT9_i1 promotes longer JNK signaling, c-Jun phosphorylation, and cyclin D1 expression, leading to enhanced proliferation (Gonzalez et al., 2009). Septins are also involved in the nucleo-cytoplasmic distribution of proteins. By binding to SOCS7, which contains the nuclear import/export signals, the SEPT2/6/7 complex maintains SOCS7 together with the adapter protein NCK in the cytoplasm, thus perturbing cell cycle arrest induced by DNA damage (Kremer et al., 2007). Also, SEPT9_i1 was evidenced to physically bind to importin-α and HIF-1α to promote HIF-1α nuclear translocation and subsequent transcriptional activation (Golan and Mabjeesh, 2013).

## Molecular determinants of septin filament localization

The high diversity of septin isoform expression and the variety of their assembly into oligomers and higher-order structures suggest a molecular basis for their multiple localizations and functions. The intimate determinants of subcellular septin fate are most often enigmatic but some targeting and/or interaction mechanisms have been described (see Figure 1).

One intriguing mechanism has been revealed in fungi and could potentially be generalized to other organisms. In polarized fungal hyphae, septins are locally translated and assembled into heteromeric complexes on the surface of shuttling endosomes for an efficient long-distance transport and deposition at growth poles (Baumann et al., 2013; Zander et al., 2016). Consistent with such a model, the majority of septin molecules found in the cytosol are already assembled into octamers, while the septin complexes recovered at the plasma membrane are composed of multiple octamers that can anneal (Bridges et al., 2014). Septin rod association into filaments would be driven by interactions with the plasma membrane (Bridges et al., 2014). Such interactions involve the binding to membrane-specific phosphoinositides like PIP2 (Zhang et al., 1999), PI4,5bis-phosphate (Bertin et al., 2010) or PI5P (Akil et al., 2016) and the local curvature of the membrane, which can be efficiently discriminated by septin rods (Bridges et al., 2016).

Septins have been found to bind actin directly (Mavrakis et al., 2014; Smith et al., 2015). The N-terminal tail of SEPT9 was recently evidenced to cross-link actin filaments by binding to three different sites on F-actin (Smith et al., 2015). While anillin has been evidenced to bridge septin filaments with actin during mitosis, BORG2 was recently shown to play a similar role in non-dividing cells (Calvo et al., 2015). BORG1, 2 and 3 were evidenced to directly bind to septin oligomers that comprise SEPT6, and this interaction is negatively regulated by Cdc42 (Joberty et al., 2001; Sadian et al., 2013). Gic1, one of the yeast homologs of human BORG, has been shown to stabilize long septin filaments by binding to the Cdc10 (a septin of the group 3) subunit (Sadian et al., 2013). In contrast, activated BORG3, by strongly binding to the SEPT6/7 interface (Sheffield et al., 2003), causes uncontrolled septin bundling and thus filament loss (Joberty et al., 2001; Kinoshita et al., 2002). More recently, BORG5 was shown to control the localization of septin filaments along the perinuclear actin fibers (Liu et al., 2014). The spatiotemporal control of septin targeting to the actin cytoskeleton may thus depend on the presence of binding partners in the cytoplasm, excluding the participation of anillin, which is sequestered in the nucleus during the interphase (Field and Alberts, 1995). Regulation of BORG availability may also exist, as BORG2 expression increased in response to cancer cell-derived soluble factors and is consistently increased in the stromal compartment of breast cancers (Calvo et al., 2015).

SEPT9, in particular SEPT9_i1, has been linked to many cancers of bad prognosis, to cell resistance to MTA and was found to be overexpressed during the interphase (G1 and S) in breast cancer (Gonzalez et al., 2009). Long SEPT9 isoforms (SEPT9_i1, i2, and i3), which are preferentially incorporated into higher-order structures than short ones (_i4-5; Sellin et al., 2011), contribute to localize septin filaments along interphase MTs (Sellin et al., 2012; Bai et al., 2013; Mizutani et al., 2013). Thus, the localization of SEPT9-containing filaments to actin in the cytoplasm and to MTs in the cilium of RPE-1 cells (Ghossoub et al., 2013), may also rely on the expression of specific *SEPT9* splice variants. By directly binding *in vitro* to MTs through its GTP-binding domain (Nagata et al., 2003), SEPT9_i1 was proposed to target septin filaments to MTs in interphase cells (Surka et al., 2002). The repeated basic motifs in the N-terminal regions of the long SEPT9 isoforms (SEPT9_i1-3) were shown to interact with the acidic regions of tubulin (Bai et al., 2013). However, a more recent study demonstrated that the whole N-terminal domain of mammalian SEPT9_i1 also contains an interaction domain with F-actin (Smith et al., 2015). These findings indicate that other molecular determinants on septins or on their target organelles may play a role in septin association with either cytoskeleton element. Tubulin posttranslational modifications are one of these determinants as highlighted by the differential septin filament locations between sensitive and Taxol®-resistant breast cancer cells, which both express long SEPT9 isoforms (Froidevaux-Klipfel et al., 2015). As already observed in MDCK cells, septin filaments coalign with polyglutamylated MT tracks (Spiliotis et al., 2008). Long lateral polyglutamate chains on tubulin even enhance this association in Taxol®-resistant breast cancer cells, while septins remain associated with actin in their Taxol®-sensitive counterpart (Froidevaux-Klipfel et al., 2015). This differential septin filament localization between MTs and actin filaments correlates with a high level of SEPT9_i1 in resistant cells, while SEPT9_i3 is the main SEPT9 isoform found in the septin filaments of sensitive cells. By bearing 5 more positive charges than SEPT9_i3, SEPT9_i1 would have more affinity for the acidic tail of tubulin, and even more when it bears long polyglutamate chains.

## Concluding remarks

Although, septin roles in cancer have been largely documented, the understanding of how the spatial and temporal dynamics of septin subcellular localization is regulated remains to be fully addressed. As described above, some clues are emerging, as the incorporation of specific SEPT9 isoforms into oligomers was found to orient the final destination of septin filaments inside the cell. Other septins are also involved, as the SEPT5-containing complexes are enriched in the lamellipodia of squamous cell carcinoma DJM-1 cells, while complexes recovered along MTs exclude this septin (Mizutani et al., 2013).

Besides filament composition, much remains to be learned about the potential roles of septin monomers vs. oligomers and vs. filaments. Indeed, while SEPT2, 7, and 11 are required at the early stages of cytokinesis, only the SEPT9 deletion impairs the final separation of daughter cells (Estey et al., 2010), suggesting a role for SEPT9 even when it is not incorporated into a filament. Also, not only filaments but septin hexamers can mediate actin bending and bundling during actin remodeling at the furrow canal in *Drosophila* embryos (Mavrakis et al., 2014). Therefore, caution must be taken when overexpressing a single septin because it might perturb the original septin filament location and/or function, as observed for some SEPT9 isoforms. Also, a strong expression of individual septins may form homo-oligomers (see Fung et al., 2014; Sellin et al., 2014) that have nothing to do with physiological septin filaments. Furthermore, posttranslational modifications (phosphorylation, acetylation, and sumoylation) may impact the way septins assemble into higher-order structures as described in fungi and will deserve further investigation in the context of cancer (Hernández-Rodriguez and Momany, 2012).

Regarding cancer therapy or cell adaptation and resistance to chemotherapies, the direct targeting of septins could have many side effects. Nevertheless, septins have been proposed as molecular targets in solid tumors, where they are required for cytokinesis completion as opposed to hematopoietic cells (Menon et al., 2014). Alternatively, research of novel therapeutic targets might focus on the perturbation of the subcellular localization of septin filaments. As such, by dampening the lateral interactions between parallel septin filaments at the anaphase spindle midline, alternating electric fields used for the treatment of recurrent glioblastoma induce mitotic catastrophe and subsequent apoptosis (Gera et al., 2015). Septin relocalization in interphase cells might as well be achieved by the targeting of their membrane- or cytoskeletal-binding partners. Understanding the underlying mechanisms of septin subcellular localization therefore deserves more interest, and will be important to focus on in the years to come.

## Author contributions

CP, LK, and AB designed and wrote the manuscript.

## Funding

This work was supported by INSERM and by Univ. Paris-Sud.

### Conflict of interest statement

The authors declare that the research was conducted in the absence of any commercial or financial relationships that could be construed as a potential conflict of interest.

## References

[B1] Ageta-Ishihara N. Miyata T. Ohshima C. Watanabe M. Sato Y. Hamamura Y.. (2013). Septins promote dendrite and axon development by negatively regulating microtubule stability via HDAC6-mediated deacetylation. Nat. Commun. 4, 2532. 10.1038/ncomms3532 24113571 PMC3826633

[B2] Akil A. Peng J. Omrane M. Gondeau C. Desterke C. Marin M. E. L.. (2016). Septin 9 induces lipid droplets growth by a phosphatidylinositol-5-phosphate and microtubule-dependent mechanism hijacked by HCV. Nat. Commun. 7, 1–19. 10.1038/ncomms12203 27417143 PMC4947189

[B3] Amir S. Mabjeesh N. J. (2007). SEPT9_V1 protein expression is associated with human cancer cell resistance to microtubule-disrupting agents. Cancer Biol. Ther. 6, 1926–1931. 10.4161/cbt.6.12.4971 18075300

[B4] Amir S. Wang R. Simons J. W. Mabjeesh N. J. (2009). SEPT9_v1 Up-regulates Hypoxia-inducible Factor 1 by Preventing Its RACK1-mediated Degradation. J. Biol. Chem. 284, 1–10. 10.1074/jbc.M808348200 19251694 PMC2670119

[B5] Bai X. Bowen J. R. Knox T. K. Zhou K. Pendziwiat M. Kuhlenbäumer G.. (2013). Novel septin 9 repeat motifs altered in neuralgic amyotrophy bind and bundle microtubules. J. Cell Biol. 203, 895–905. 10.1083/jcb.201308068 24344182 PMC3871440

[B6] Barve G. Sridhar S. Aher A. Singh S. Lakshmeesha K. N. Manjithaya R. (2016). Septins are involved at the early stages of macroautophagy. BioRXiv. 10.1101/043133. [Epub ahead of print]. PMC5868950 29361537

[B7] Baumann S. König J. Koepke J. Feldbrügge M. (2013). Endosomal transport of septin mRNA and protein indicates local translation on endosomes and is required for correct septin filamentation. EMBO Rep. 15, 94–102. 10.1002/embr.201338037 24355572 PMC4303453

[B8] Bertin A. McMurray M. A. Thai L. Garcia G. Votin V. Grob P.. (2010). Phosphatidylinositol-4,5-bisphosphate promotes budding yeast septin filament assembly and organization. J. Mol. Biol. 404, 711–731. 10.1016/j.jmb.2010.10.002 20951708 PMC3005623

[B9] Bowen J. R. Hwang D. Bai X. Roy D. Spiliotis E. T. (2011). Septin GTPases spatially guide microtubule organization and plus end dynamics in polarizing epithelia. J. Cell Biol. 194, 187–197. 10.1083/jcb.201102076 21788367 PMC3144415

[B10] Bridges A. A. Jentzsch M. S. Oakes P. W. Occhipinti P. Gladfelter A. S. (2016). Micron-scale plasma membrane curvature is recognized by the septin cytoskeleton. J. Cell Biol. 213, 23–32. 10.1083/jcb.201512029 27044896 PMC4828694

[B11] Bridges A. A. Zhang H. Mehta S. B. Occhipinti P. Tani T. Gladfelter A. S. (2014). Septin assemblies form by diffusion-driven annealing on membranes. Proc. Natl. Acad. Sci. U.S.A. 111, 2146–2151. 10.1073/pnas.1314138111 24469790 PMC3926015

[B12] Calvo F. Ranftl R. Hooper S. Farrugia A. J. Moeendarbary E. Bruckbauer A.. (2015). Cdc42EP3/BORG2 and septin network enables mechano-transduction and the emergence of cancer-associated fibroblasts. Cell Rep. 13, 2699–2714. 10.1016/j.celrep.2015.11.052 26711338 PMC4700053

[B13] Caudron F. Barral Y. (2009). Septins and the lateral compartmentalization of eukaryotic membranes. Dev. Cell 16, 493–506. 10.1016/j.devcel.2009.04.003 19386259

[B14] Cerveira N. Bizarro S. Teixeira M. R. (2011). MLL-SEPTIN gene fusions in hematological malignancies. Biol. Chem. 392, 1–12. 10.1515/BC.2011.072 21714766

[B15] Chacko A. D. Hyland P. L. McDade S. S. Hamilton P. W. Russell S. H. Hall P. A. (2005). SEPT9_v4 expression induces morphological change, increased motility and disturbed polarity. J. Pathol. 206, 458–465. 10.1002/path.1794 15902694

[B16] Chacko A. D. McDade S. S. Chanduloy S. Church S. W. Kennedy R. Price J.. (2012). Expression of the SEPT9_i4 isoform confers resistance to microtubule-interacting drugs. Cell. Oncol. 35, 85–93. 10.1007/s13402-011-0066-0 22278362 PMC12995059

[B17] Connolly D. Hoang H. G. Adler E. Tazearslan C. Simmons N. Bernard V. V.. (2014). Septin 9 amplification and isoform-specific expression in peritumoral and tumor breast tissue. Biol. Chem. 395, 157–167. 10.1515/hsz-2013-0247 24127542

[B18] Connolly D. Yang Z. Castaldi M. Simmons N. Oktay M. H. Coniglio S.. (2011). Septin 9 isoform expression, localization and epigenetic changes during human and mouse breast cancer progression. Breast Cancer Res. 13, R76. 10.1186/bcr2924 21831286 PMC3236340

[B19] Cortez B. A. Rezende-Teixeira P. Redick S. Doxsey S. Machado-Santelli G. M. (2016). Multipolar mitosis and aneuploidy after chrysotile treatment: a consequence of abscission failure and cytokinesis regression. Oncotarget 7, 8979–8992. 10.18632/oncotarget.6924 26788989 PMC4891019

[B20] Cui C. Chatterjee B. Lozito T. P. Zhang Z. Francis R. J. Yagi H.. (2013). Wdpcp, a PCP protein required for ciliogenesis, regulates directional cell migration and cell polarity by direct modulation of the actin cytoskeleton. PLoS Biol. 11:e1001720. 10.1371/journal.pbio.1001720 24302887 PMC3841097

[B21] Diesenberg K. Beerbaum M. Fink U. Schmieder P. Krauss M. (2015). SEPT9 negatively regulates ubiquitin-dependent downregulation of EGFR. J. Cell Sci. 128, 397–407. 10.1242/jcs.162206 25472714

[B22] Dolat L. Hunyara J. L. Bowen J. R. Karasmanis E. P. Elgawly M. Galkin V. E.. (2014). Septins promote stress fiber-mediated maturation of focal adhesions and renal epithelial motility. J. Cell Biol. 207, 225–235. 10.1083/jcb.201405050 25349260 PMC4210437

[B23] Edison N. Reingewertz T.-H. Gottfried Y. Lev T. Zuri D. Maniv I.. (2012). Peptides mimicking the unique ARTS-XIAP binding site promote apoptotic cell death in cultured cancer cells. Clin. Cancer Res. 18, 2569–2578. 10.1158/1078-0432.CCR-11-1430 22392914

[B24] Elhasid R. Larisch S. (2011). ARTS-based anticancer therapy: taking aim at cancer stem cells. Future Oncol. 7, 1185–1194. 10.2217/fon.11.96 21992730

[B25] Estey M. P. Di Ciano-Oliveira C. Froese C. D. Bejide M. T. Trimble W. S. (2010). Distinct roles of septins in cytokinesis: SEPT9 mediates midbody abscission. J. Cell Biol. 191, 741–749. 10.1083/jcb.201006031 21059847 PMC2983063

[B26] Estey M. P. Di Ciano-Oliveira C. Froese C. D. Fung K. Y. Y. Steels J. D. Litchfield D. W.. (2013). Mitotic regulation of SEPT9 protein by cyclin-dependent kinase 1 (Cdk1) and pin1 protein is important for the completion of cytokinesis. J. Biol. Chem. 288, 30075–30086. 10.1074/jbc.M113.474932 23990466 PMC3798476

[B27] Field C. M. Alberts B. M. (1995). Anillin, a contractile ring protein that cycles from the nucleus to the cell cortex. J. Cell Biol. 131, 165–178. 10.1083/jcb.131.1.165 7559773 PMC2120607

[B28] Froidevaux-Klipfel L. Poirier F. Boursier C. Crépin R. Poüs C. Baudin B.. (2011). Modulation of septin and molecular motor recruitment in the microtubule environment of the Taxol-resistant human breast cancer cell line MDA-MB-231. Proteomics 11, 3877–3886. 10.1002/pmic.201000789 21761557

[B29] Froidevaux-Klipfel L. Targa B. Cantaloube I. Ahmed-Zaïd H. Poüs C. Baillet A. (2015). Septin cooperation with tubulin polyglutamylation contributes to cancer cell adaptation to taxanes. Oncotarget 6, 36063–36080. 10.18632/oncotarget.5373 26460824 PMC4742162

[B30] Füchtbauer A. Lassen L. B. Jensen A. B. Howard J. Quiroga Ade S. Warming S.. (2011). Septin9 is involved in septin filament formation and cellular stability. Biol. Chem. 392, 769–777. 10.1515/BC.2011.088 21824004

[B31] Fung K. Y. Dai L. Trimble W. S. (2014). Cell and molecular biology of septins. Int. Rev. Cell Mol. Biol. 310, 289–339. 10.1016/B978-0-12-800180-6.00007-4 24725429

[B32] García-Fernández M. Kissel H. Brown S. Gorenc T. Schiele A. J. Rafii S.. (2010). *SEPT4/ARTS* is required for stem cell apoptosis and tumor suppression. Genes Dev. 24, 2282–2293. 10.1101/gad.1970110 20952537 PMC2956207

[B33] Gera N. Yang A. Holtzman T. S. Lee S. X. Wong E. T. Swanson K. D. (2015). Tumor treating fields perturb the localization of septins and cause aberrant mitotic exit. PLoS ONE 10:e0125269. 10.1371/journal.pone.0125269 26010837 PMC4444126

[B34] Ghossoub R. Hu Q. Failler M. Rouyez M. C. Spitzbarth B. Mostowy S.. (2013). Septins 2, 7 and 9 and MAP4 colocalize along the axoneme in the primary cilium and control ciliary length. J. Cell Sci. 126, 2583–2594. 10.1242/jcs.111377 23572511 PMC3687695

[B35] Gilad R. Meir K. Stein I. German L. Pikarsky E. Mabjeesh N. J. (2015). High SEPT9_i1 protein expression is associated with high-grade prostate cancers. PLoS ONE 10:e0124251. 10.1371/journal.pone.0124251 25898316 PMC4405336

[B36] Golan M. Mabjeesh N. J. (2013). SEPT9_i1 is required for the association between HIF-1α and importin-α to promote efficient nuclear translocation. Cell Cycle 12, 2297–2308. 10.4161/cc.25379 24067372 PMC3755080

[B37] Gonzalez M. E. Makarova O. Peterson E. A. Privette L. M. Petty E. M. (2009). Up-regulation of SEPT9_v1 stabilizes c-Jun-N-Terminal kinase and contributes to its pro-proliferative activity in mammary epithelial cells. Cell. Signal. 21, 477–487. 10.1016/j.cellsig.2008.11.007 19071215 PMC2811713

[B38] Hagiwara A. Tanaka Y. Hikawa R. Morone N. Kusumi A. Kimura H.. (2011). Submembranous septins as relatively stable components of actin-based membrane skeleton. Cytoskeleton (Hoboken) 68, 512–525. 10.1002/cm.20528 21800439

[B39] Hall P. A. Russell S. E. H. (2012). Mammalian septins: dynamic heteromers with roles in cellular morphogenesis and compartmentalization. J. Pathol. 226, 287–299. 10.1002/path.3024 21990096

[B40] Hall P. A. Todd C. B. Hyland P. L. McDade S. S. Grabsch H. Dattani M.. (2005). The septin-binding protein anillin is overexpressed in diverse human tumors. Clin. Cancer Res. 11, 6780–6786. 10.1158/1078-0432.CCR-05-0997 16203764

[B41] Hernández-Rodriguez Y. Momany M. (2012). Posttranslational modifications and assembly of septin heteropolymers and higher-order structures. Curr. Opin. Microbiol. 15, 660–668. 10.1016/j.mib.2012.09.007 23116980

[B42] Hu Q. Nelson W. J. (2011). Ciliary diffusion barrier: the gatekeeper for the primary cilium compartment. Cytoskeleton (Hoboken) 68, 313–324. 10.1002/cm.20514 21634025 PMC3143192

[B43] Joberty G. Perlungher R. R. Sheffield P. J. Kinoshita M. Noda M. Haystead T.. (2001). Borg proteins control septin organization and are negatively regulated by Cdc42. Nat. Cell Biol. 3, 861–866. 10.1038/ncb1001-861 11584266

[B44] Kinoshita M. (2003). Assembly of mammalian septins. J. Biochem. 134, 491–496. 10.1093/jb/mvg182 14607974

[B45] Kinoshita M. Field C. M. Coughlin M. L. Straight A. F. Mitchison T. J. (2002). Self- and actin-templated assembly of mammalian septins. Dev. Cell 3, 1–12. 10.1016/S1534-5807(02)00366-0 12479805

[B46] Kinoshita M. Kumar S. Mizoguchi A. Ide C. Kinoshita A. Haraguchi T.. (1997). Nedd5, a mammalian septin, is a novel cytoskeletal component interacting with actin-based structures. Genes Dev. 11, 1535–1547. 10.1101/gad.11.12.1535 9203580

[B47] Kremer B. E. Adang L. A. MacAra I. G. (2007). Septins regulate actin organization and cell-cycle arrest through nuclear accumulation of NCK mediated by SOCS7. Cell 130, 837–850. 10.1016/j.cell.2007.06.053 17803907 PMC2085444

[B48] Kremer B. E. Haystead T. MacAra I. G. (2005). Mammalian septins regulate microtubule stability through interaction with the microtubule-binding protein MAP4. Mol. Biol. Cell 16, 4648–4659. 10.1091/mbc.E05-03-0267 16093351 PMC1237071

[B49] Liu M. Shen S. Chen F. Yu W. Yu L. (2010). Linking the septin expression with carcinogenesis. Mol. Biol. Rep. 37, 3601–3608. 10.1007/s11033-010-0009-2 20195767

[B50] Liu Z. Vong Q. P. Liu C. Zheng Y. (2014). Borg5 is required for angiogenesis by regulating persistent directional migration of the cardiac microvascular endothelial cells. Mol. Biol. Cell 25, 841–851. 10.1091/mbc.E13-09-0543 24451259 PMC3952853

[B51] Marcus E. A. Tokhtaeva E. Turdikulova S. Capri J. Whitelegge J. P. Scott D. R.. (2016). Septin oligomerization regulates persistent expression of ErbB2/HER2 in gastric cancer cells. Biochem. J. 473, 1703–1718. 10.1042/BCJ20160203 27048593 PMC4903893

[B52] Mavrakis M. Azou-Gros Y. Tsai F.-C. Alvarado J. Bertin A. Iv F.. (2014). Septins promote F-actin ring formation by crosslinking actin filaments into curved bundles. Nat. Cell Biol. 16, 322–334. 10.1038/ncb2921 24633326

[B53] Menon M. B. Gaestel M. (2015). Sep(t)arate or not - how some cells take septin-independent routes through cytokinesis. J. Cell Sci. 128, 1877–1886. 10.1242/jcs.164830 25690008

[B54] Menon M. B. Sawada A. Chaturvedi A. Mishra P. Schuster-Gossler K. Galla M.. (2014). Genetic deletion of SEPT7 reveals a cell type-specific role of septins in microtubule destabilization for the completion of cytokinesis. PLoS Genet. 10:e1004558. 10.1371/journal.pgen.1004558 25122120 PMC4133155

[B55] Mizutani Y. Ito H. Iwamoto I. Morishita R. Kanoh H. Seishima M.. (2013). Possible role of a septin, SEPT1, in spreading in squamous cell carcinoma DJM-1 cells. Biol. Chem. 394, 281–290. 10.1515/hsz-2012-0258 23087102

[B56] Montagna C. Sagie M. Zechmeister J. (2015). Mammalian septins in health and disease. Res. Rep. Biochem. 5, 59–14. 10.2147/RRBC.S59060

[B57] Mostowy S. Cossart P. (2012). Septins: the fourth component of the cytoskeleton. Nat. Rev. Mol. Cell. Biol. 13, 183–194. 10.1038/nrm3284 22314400

[B58] Mostowy S. Janel S. Forestier C. Roduit C. Kasas S. Pizarro-Cerdá J.. (2011). A role for septins in the interaction between the listeria monocytogenes invasion protein InlB and the met receptor. Biophys. J. 100, 1949–1959. 10.1016/j.bpj.2011.02.040 21504731 PMC3077699

[B59] Nagata K. I. Kawajiri A. Matsui S. Takagishi M. Shiromizu T. Saitoh N.. (2003). Filament formation of MSF-A, a mammalian septin, in human mammary epithelial cells depends on interactions with microtubules. J. Biol. Chem. 278, 18538–18543. 10.1074/jbc.M205246200 12626509

[B60] Osaka M. Rowley J. D. Zeleznik-Le N. J. (1999). MSF (MLL septin-like fusion), a fusion partner gene of MLL, in a therapy-related acute myeloid leukemia with a t(11;17)(q23;q25). Proc. Natl. Acad. Sci. U.S.A. 96, 6428–6433. 10.1073/pnas.96.11.6428 10339604 PMC26898

[B61] Pagliuso A. Tham T. N. Stevens J. K. Lagache T. Persson R. Salles A.. (2016). A role for septin 2 in Drp1-mediated mitochondrial fission. EMBO Rep. 17, 858–873. 10.15252/embr.201541612 27215606 PMC5278612

[B62] Renshaw M. J. Liu J. Lavoie B. D. Wilde A. (2014). Anillin-dependent organization of septin filaments promotes intercellular bridge elongation and Chmp4B targeting to the abscission site. Open Biol. 4, 130190–130190. 10.1098/rsob.130190 24451548 PMC3909275

[B63] Rocha C. Papon L. Cacheux W. Marques Sousa P. Lascano V. Tort O.. (2014). Tubulin glycylases are required for primary cilia, control of cell proliferation and tumor development in colon. EMBO J. 33, 2247–2260. 10.15252/embj.201488466 25180231 PMC4282510

[B64] Saarikangas J. Barral Y. (2011). The emerging functions of septins in metazoans. EMBO Rep. 12, 1118–1126. 10.1038/embor.2011.193 21997296 PMC3207108

[B65] Sadian Y. Gatsogiannis C. Patasi C. Hofnagel O. Goody R. S. Farkašovský M.. (2013). The role of Cdc42 and Gic1 in the regulation of septin filament formation and dissociation. eLife 2, 8274–8226. 10.7554/eLife.01085 24286829 PMC3840788

[B66] Schmidt K. Nichols B. J. (2004). Functional interdependence between septin and actin cytoskeleton. BMC Cell Biol. 5:43. 10.1186/1471-2121-5-43 15541171 PMC535351

[B67] Scott M. McCluggage W. G. Hillan K. J. Hall P. A. Russell S. E. H. (2006). Altered patterns of transcription of the septin gene, SEPT9, in ovarian tumorigenesis. Int. J. Cancer 118, 1325–1329. 10.1002/ijc.21486 16161048

[B68] Sellin M. E. Sandblad L. Stenmark S. Gullberg M. (2011). Deciphering the rules governing assembly order of mammalian septin complexes. Mol. Biol. Cell 22, 3152–3164. 10.1091/mbc.E11-03-0253 21737677 PMC3164462

[B69] Sellin M. E. Stenmark S. Gullberg M. (2012). Mammalian SEPT9 isoforms direct microtubule-dependent arrangements of septin core heteromers. Mol. Biol. Cell 23, 4242–4255. 10.1091/mbc.E12-06-0486 22956766 PMC3484102

[B70] Sellin M. E. Stenmark S. Gullberg M. (2014). Cell type-specific expression of SEPT3-homology subgroup members controls the subunit number of heteromeric septin complexes. Mol. Biol. Cell 25, 1594–1607. 10.1091/mbc.E13-09-0553 24648497 PMC4019491

[B71] Shankar J. Messenberg A. Chan J. Underhill T. M. Foster L. J. Nabi I. R. (2010). Pseudopodial actin dynamics control epithelial-mesenchymal transition in metastatic cancer cells. Cancer Res. 70, 3780–3790. 10.1158/0008-5472.CAN-09-4439 20388789

[B72] Sheffield P. J. Oliver C. J. Kremer B. E. Sheng S. Shao Z. MacAra I. G. (2003). Borg/septin interactions and the assembly of mammalian septin heterodimers, trimers, and filaments. J. Biol. Chem. 278, 3483–3488. 10.1074/jbc.M209701200 12446710

[B73] Shen S. Liu M. Wu Y. Saiyin H. Liu G. Yu L. (2012). Involvement of SEPT4_i1 in hepatocellular carcinoma: SEPT4_i1 regulates susceptibility to apoptosis in hepatocellular carcinoma cells. Mol. Biol. Rep. 39, 4519–4526. 10.1007/s11033-011-1242-z 21952823

[B74] Silverman-Gavrila R. Silverman-Gavrila L. (2008). Septins: new microtubule interacting partners. Sci. World J. 8, 611–620. 10.1100/tsw.2008.87 18604445 PMC5848705

[B75] Sirianni A. Krokowski S. Lobato-Márquez D. Buranyi S. Pfanzelter J. Galea D.. (2016). Mitochondria mediate septin cage assembly to promote autophagy of Shigella. EMBO Rep. 17, 1029–1043. 10.15252/embr.201541832 27259462 PMC4931556

[B76] Smith C. Dolat L. Angelis D. Forgacs E. Spiliotis E. T. Galkin V. E. (2015). Septin 9 exhibits polymorphic binding to f-actin and inhibits myosin and cofilin activity. J. Mol. Biol. 427, 3273–3284. 10.1016/j.jmb.2015.07.026 26297986 PMC4587343

[B77] Song L. Li Y. (2015). SEPT9: a specific circulating biomarker for colorectal cancer. Adv. Clin. Chem. 72, 171–204. 10.1016/bs.acc.2015.07.004 26471083

[B78] Spiliotis E. T. Gladfelter A. S. (2012). Spatial guidance of cell asymmetry: septin GTPases show the way. Traffic 13, 195–203. 10.1111/j.1600-0854.2011.01268.x 21883761 PMC3245886

[B79] Spiliotis E. T. Hunt S. J. Hu Q. Kinoshita M. Nelson W. J. (2008). Epithelial polarity requires septin coupling of vesicle transport to polyglutamylated microtubules. J. Cell Biol. 180, 295–303. 10.1083/jcb.200710039 18209106 PMC2213583

[B80] Spiliotis E. T. Kinoshita M. Nelson W. J. (2005). A mitotic septin scaffold required for Mammalian chromosome congression and segregation. Science 307, 1781–1785. 10.1126/science.1106823 15774761 PMC3368603

[B81] Surka M. C. Tsang C. W. Trimble W. S. (2002). The mammalian septin MSF localizes with microtubules and is required for completion of cytokinesis. Mol. Biol. Cell 13, 3532–3545. 10.1091/mbc.E02-01-0042 12388755 PMC129964

[B82] Tanaka-Takiguchi Y. Kinoshita M. Takiguchi K. (2009). Septin-mediated uniform bracing of phospholipid membranes. Curr. Biol. 19, 140–145. 10.1016/j.cub.2008.12.030 19167227

[B83] Tooley A. J. Gilden J. Jacobelli J. Beemiller P. Trimble W. S. Kinoshita M.. (2009). Amoeboid T lymphocytes require the septin cytoskeleton for cortical integrity and persistent motility. Nat. Cell Biol. 11, 17–26. 10.1038/ncb1808 19043408 PMC3777658

[B84] Xu M. Takanashi M. Oikawa K. Nishi H. Isaka K. Yoshimoto T.. (2012). Identification of a novel role of Septin 10 in paclitaxel-resistance in cancers through a functional genomics screen. Cancer Sci. 103, 821–827. 10.1111/j.1349-7006.2012.02221.x 22320903 PMC7659246

[B85] Yeh Y.-T. Hur S. S. Chang J. Wang K.-C. Chiu J.-J. Li Y.-S.. (2012). Matrix stiffness regulates endothelial cell proliferation through septin 9. PLoS ONE 7:e46889. 10.1371/journal.pone.0046889 23118862 PMC3485289

[B86] Zander S. Baumann S. Weidtkamp-Peters S. Feldbrügge M. (2016). Endosomal assembly and transport of heteromeric septin complexes promote septin cytoskeleton formation. J. Cell Sci. 129, 2778–2792. 10.1242/jcs.182824 27252385

[B87] Zhang J. Kong C. Xie H. McPherson P. S. Grinstein S. Trimble W. S. (1999). Phosphatidylinositol polyphosphate binding to the mammalian septin H5 is modulated by GTP. Curr. Biol. 9, 1458–1467. 10.1016/S0960-9822(00)80115-3 10607590

[B88] Zhou X. Z. Lu K. P. (2016). The isomerase PIN1 controls numerous cancer-driven pathways and is a unique drug target. Nat. Rev. Cancer 16, 463–478. 10.1038/nrc.2016.49v 27256007

